# Expansion of the Genotypic and Phenotypic Spectrum of *ASH1L*-Related Syndromic Neurodevelopmental Disorder

**DOI:** 10.3390/genes15040423

**Published:** 2024-03-28

**Authors:** Ineke Cordova, Alyssa Blesson, Juliann M. Savatt, Abigail Sveden, Sonal Mahida, Heather Hazlett, Erin Rooney Riggs, Maya Chopra

**Affiliations:** 1 Autism and Developmental Medicine Institute, Geisinger, Danville, PA 17822, USA; icordova@geisinger.edu (I.C.); eriggs@geisinger.edu (E.R.R.); 2 Department of Neurology and Developmental Medicine, Kennedy Krieger Institute, Baltimore, MD 21205, USA; 3 Rosamund Stone Zander Translational Neuroscience Center, Boston Children’s Hospital, Boston, MA 02115, USA; 4 Department of Psychiatry, University of North Carolina Intellectual and Developmental Disability Research Center, Chapel Hill, NC 27510, USA

**Keywords:** *ASH1L*, neurodevelopmental disorder, histone methyltransferase, autism spectrum disorder, intellectual disability, syndromic complex neurodevelopmental disorder, Brain Gene Registry, GenomeConnect, ClinGen

## Abstract

Pathogenic *ASH1L* variants have been reported in probands with broad phenotypic presentations, including intellectual disability, autism spectrum disorder, attention deficit hyperactivity disorder, seizures, congenital anomalies, and other skeletal, muscular, and sleep differences. Here, we review previously published individuals with pathogenic *ASH1L* variants and report three further probands with novel *ASH1L* variants and previously unreported phenotypic features, including mixed receptive language disorder and gait disturbances. These novel data from the Brain Gene Registry, an accessible repository of clinically derived genotypic and phenotypic data, have allowed for the expansion of the phenotypic and genotypic spectrum of this condition.

## 1. Introduction

*ASH1L*, located at chromosomal band 1q22, encodes a histone methyltransferase that catalyzes histone H3K36 methylation and associates with “most, if not all, active genes” [1,2]. It also has a role in chromatin modification and gene transcription [3,4]. Heterozygous pathogenic variants in *ASHL1* have been implicated in an autosomal dominant neurodevelopmental disorder (NDD). The range of neurodevelopmental features observed in individuals with pathogenic variants in *ASH1L* is broad and includes intellectual disability (ID) and autism spectrum disorder (ASD), with variable presence of congenital anomalies and non-specific dysmorphic features [5,6,7,8,9]. To date, accounts of only 11 individuals with heterozygous loss-of-function (LOF) variants (nonsense, frameshift, and deletion) in *ASH1L* have been published with detailed phenotyping, limiting understanding of the full phenotypic spectrum of the disorder. Considering the reported impact of truncating and nonsense *ASH1L* variants in both humans and mice and the intolerance to heterozygous LOF in the general population (pLI = 1 and pLoF = 125.3, [10]), haploinsufficiency is believed to be at least one disease mechanism [9,11,12]. Reportedly disease-causing missense variants have also been described in the literature, and some have asserted that these patients’ phenotypes are more severe than those with LoF variants. However, additional functional studies are needed to understand the mechanism of disease for missense variants [11]

*ASH1L* variants were first implicated in human disease in 2012 through a large sequencing study in persons with severe ID. Six rare, *de novo* variants in probands with ID were reported, but detailed clinical information was limited to only one proband [8]. Subsequently, a more complex and syndromic phenotypic spectrum has emerged. In addition to DD/ID and ASD, genital and urinary malformations (such as penile torsion and cryptorchidism) and sleep disorders have also been reported. A smaller percentage of probands also have been reported with seizures, feeding difficulties, hypotonia, musculoskeletal abnormalities (pectus carinatum, pectus excavatum, scoliosis, and cervical spinal fusion), ophthalmological abnormalities (hyperopia, astigmatism, strabismus, and nystagmus), hearing impairments, and gastrointestinal disturbances [11,13,14,15,16]. Variable dysmorphic facial features have also been reported but with no determinable pattern [11,13,14,15] (Table S1). Considering the variable syndromic phenotypic features reported, the ClinGen Intellectual Disability and Autism Gene Curation Expert Panel curated *ASH1L* in relationship to syndromic complex neurodevelopmental disorder (ClinGen Intellectual Disability and Autism GCEP 2023) [17,18]. Based on the available genetic and experimental evidence, including mouse models [12,19,20], the gene’s relationship to syndromic complex neurodevelopmental disorder was classified as Definitive.

Understanding the prevalence and breadth of the phenotypic spectrum for conditions like *ASH1L*-related disorder poses challenges. To date, the primary way by which information about the phenotypic spectrum of a disorder is disseminated is through the literature. However, once the seminal paper asserting a relationship between the disorder and variation in a particular gene is published, it is often difficult to publish subsequent case reports due to perceived lack of novelty, thus limiting our understanding of the full phenotypic spectrum. Additional observations of variants within the gene may be publicly available in databases such as ClinVar, but these submissions often lack detailed clinical information. Even when phenotypes are reported both in the literature and in ClinVar, subclinical phenotypes are often missed or ignored. 

The Brain Gene Registry (BGR) is a consortium of 13 Intellectual and Disability Research Centers (IDDRCs) established to better understand gene–disease relationships, creating an accessible repository of clinically derived, paired genotypic and phenotypic data taken from a standardized neurobehavioral battery and the electronic health record (https://braingeneregistry.wustl.edu/ (accessed on 17 August 2023)) [21]. Here, we summarize prior reports of patients with *ASH1L* variants and report detailed analyses on three BGR participants with novel variants and previously unreported phenotypic features.

## 2. Materials and Methods

### Recruitment

Individuals were co-enrolled in the BGR and GenomeConnect [22,23], the ClinGen patient registry. Details of recruitment and co-enrollment methodologies between the BGR and GenomeConnect have been previously described [21]. Briefly, eligibility is determined by the presence of clinically ascertained variants (with an ACMG/AMP classification of variant of uncertain significance (VUS) and above) in any gene implicated in neurodevelopment. Through the BGR, the participant then completes the BGR’s virtual Rapid Neurobehavioral Assessment Protocol (RNAP), questionnaires, behavioral surveys, and a telehealth assessment (including dysmorphology screening). The BGR then extrapolates additional phenotype data from the RNAP and from the electronic health record (EHR) to provide a standardized phenotypic assessment for each participant. GenomeConnect enrolls anyone who has received genetic testing regardless of result or diagnosis. BGR participants are directed to co-enroll in GenomeConnect to enable collection of structured genomic data and patient-derived phenotypes and allow de-identified data sharing with databases including NCBI’s ClinVar, and, in the case of candidate genes, MatcherMaker Exchange [24,25]. Enrollment in GenomeConnect also allows participants the opportunity to be recontacted for future research, to connect with others with similar results, and to receive updates about their genetic results.

After co-enrollment into BGR and GenomeConnect, those with clinically identified *ASH1L* variants as of 24 October 2023 were reviewed and included in the present study if they harbored no other likely pathogenic or pathogenic variants potentially causative of their neurodevelopmental phenotypes and their variants were presumed to be loss-of-function. The BGR and GenomeConnect enrolled three participants with missense variants classified as VUS, but additional data are needed to determine their disease mechanism and pathogenicity and are, therefore, not included in this publication. Phenotypic data were extrapolated both from the BGR and GenomeConnect’s parent-completed health surveys. Given the ability of GenomeConnect to recontact participants, we contacted participants to specifically inquire about the presence or absences of phenotypes reported in other *ASH1L* participants and/or the literature but not otherwise remarked on in the standard clinical information available to the BGR.

All probands underwent different testing methodologies due to the nature of the BGR’s recruitment strategy (i.e., clinical testing in a variety of different contexts via multiple sites). *BGR 1*—The participant’s variant was first identified through an exome study. Clinical sequencing of *ASH1L* then confirmed presence of the variant in the proband and its *de novo* inheritance. *BGR 2*—Clinical trio-based exome sequencing was performed, identifying the *ASH1L* variant, followed by trio research-based genome sequencing which also identified additional variants in *TSHZ3* and *TNR*. *BGR 3*—Clinical proband-only exome sequencing was performed. To learn more about the workflow for this study and for the BGR, please see Chopra et al., 2023 [21].

## 3. Results

Ten LoF and four missense variants have been reported in the literature with detailed phenotypic information (Table S1). After internal review, phenotypes from Wang et al.’s 2016 proband were not included in the summary table due to the high frequency of the variant in gnomAD [5,10]. The BGR probands with novel LoF variants presented with both previously reported and unreported phenotypes (Table 1). Consistent with previous probands, most BGR participants reported ID/DD, ASD, obsessive behaviors, speech and motor delays, seizures, ADHD, gastrointestinal disturbances, and/or anxiety. 

### 3.1. BGR 1

The first proband, BGR 1, was enrolled into the BGR at 14 years old and is male with a de novo, predicted null variant (c.1603G > T, p.Gly535*; NM_018489.3, ClinVar SCV—SCV003927994.1) classified as likely pathogenic by the reporting laboratory in 2020 [26]. His features are consistent with individuals with previously reported ASH1L variants, including ID ((DP4 = 80; Shipley = 79) [27] and global adaptive (Vineland Adaptive Behavior Scales (VABS) = 76)) and speech delays (first words at 18 months) [28], febrile seizures beginning at age 3, ASD, attention deficit hyperactivity disorder (ADHD), obsessive behaviors, gastroesophageal reflux disease, and astigmatism. This proband also has features which have not been previously reported, including depression (diagnosed at age 11) and mixed receptive language disorder (diagnosed at age 7). Of note, this proband does not have features reported in other probands such as genital malformations, motor delay, feeding difficulties, hypotonia, skeletal malformations, or hearing impairments. The proband is reported to be non-dysmorphic. Family history is significant for dyslexia and autistic features on the paternal side of the family.

### 3.2. BGR 2

The second proband, BGR 2, was enrolled into the BGR at 8 years old and is male. BGR 2 has a de novo frameshift variant (c.4902_4903del, p.Ser1635Cysfs*18; NM_018489.3, ClinVar SCV—SCV003927995.1) classified as pathogenic by the reporting laboratory in 2019 [26]. His neurodevelopmental phenotype is consistent with previous publications including ASD, ADHD, obsessive behaviors, feeding difficulties, and excessive reflux and vomiting during infancy. The BGR’s RNAP performed at age 8 indicated average cognitive functioning (no ID) based on Shipley score (101) [27], and elevated behavioral ratings suggest ADHD and ASD symptoms (from Social Responsiveness Scale and Child Behavior Checklist data) [29,30,31] plus low adaptive functioning (from VABS) [28]. In addition, the proband displays dysmetria and an abnormal, ataxic gait with difficulty coordinating motion and balance. This proband does not have phenotypes reported in other probands such as seizures, hypotonia, genital malformations, sleep disorders, skeletal malformations, or ocular issues. The proband is reported to be non-dysmorphic. Of note, after clinical testing, this proband entered a research study which identified three VUS. One variant was reported in TSHZ3, a gene which has no disease assertions in OMIM. Biallelic variants were also found in TNR, which has been reported in relation to autosomal recessive non-progressive neurodevelopmental disorder with spasticity and transient opisthotonos (MIM#619653) [32].

### 3.3. BGR 3

The third proband, BGR 3, was enrolled into the BGR at age 15 years and is male. BGR 3 has a predicted null variant of unknown inheritance (c.4909C>T, p.Gln1637*; NM_018489.3, ClinVar SCV—SCV003935025.1) classified as likely pathogenic by the testing laboratory in 2020 [26]. This proband has a score of 75 on the Developmental Profile 4 (DP™-4), indicating borderline cognitive functioning [33], ASD, mild bilateral sensorineural hearing loss, and epilepsy (absence, focal motor, and myoclonic seizures); this proband’s epilepsy is Klonopin-resistant. The proband also has a stiff gait with left internal tibial torsion and is now walking without assistance. This proband does not have phenotypes reported in other probands such as sleep disorders or ocular issues. Of note, the proband also has three VUSs identified. The first is a missense variant in MED12 which in OMIM has disease assertions with four X-linked syndromic intellectual disability syndromes: Hardikar syndrome (MIM# 301068), Lujan–Fryns syndrome (MIM# 309520), Ohdo syndrome (MIM# 300895), and Opitz–Kaveggia syndrome (MIM# 305450). The second is a missense variant in POU4F3 which is reported to have associations with deafness: autosomal dominant 15 (MIM# 602459). The last is a missense variant in ATP2B2 which has the disease assertion of deafness: autosomal dominant 82 (MIM# 619804) [32]. The family history is significant for biological parents who reportedly share some of the features included in the clinical indication for testing of this proband; however, this proband is adopted, and no further information is available on his biological family. 

## 4. Discussion

Like many NDDs, *ASH1L*-related SCND had been previously reported with many complex phenotypes (Table S1). 

Individuals with *ASH1L* variants present with a broad spectrum of neurodevelopmental disorders. ID/DD is the most frequent phenotype (15/16, 94%), with other common phenotypes being ADHD or hyperactivity (6/7, 86%), obsessive behaviors (4/5, 80%), gastrointestinal disturbances (6/8, 75%), facial dysmorphisms (9/13, 69%), sleep disorders (6/9, 67%), ASD (6/10, 60%), ocular problems (6/10, 60%), hypotonia (4/7, 57%), genital malformations (5/9, 56%), speech delay (6/10, 60%), seizures (6/11, 55%), motor delay (6/12, 50%), feeding difficulties (4/8, 50%), mood disorders (2/4, 50%), and abnormal gait (2/4, 50%); other less common phenotypes include hearing impairments (4/9, 44%), pectus carinatum/excavatum (2/7, 29%), and scoliosis (2/7, 29%) [6,8,11,13,14,15,16]. Although most reported probands are male, there are no notable sex differences in the presentation of the condition at this time.

The BGR probands expanded our understanding of the phenotypic presentation of *ASH1L*-related disorder. BGR 1 presents with the previously unreported phenotypes of depression (diagnosed at age 11) and mixed receptive language disorder (diagnosed at age 7). Most of BGR 2’s neurodevelopmental phenotype is consistent with previous publications including ASD and ADHD. However, BGR 2 is the first proband whose intelligence and development fell within a standard range (Shipley score (101)) but reported additional features consistent with a complex neurodevelopmental condition. Additionally, BGR 2 and 3 are the first probands reported with abnormal gait. BGR 2 originally received clinical exome sequencing which reported the *ASH1L* variant as the only clinically significant variant. Subsequently, they were enrolled in research genome sequencing revealing biallelic missense variants in *TNR,* classified as VUSs. While *TNR* has been implicated in autosomal recessive neurodevelopmental disorder, non-progressive, with spasticity and transient opisthotonos (MIM#619653) (Online Mendelian Inheritance in Man, OMIM^®^), bi-allelic LoF is the presumed mechanism, and these variants are missense. At this time, these variants have not been clinically confirmed and the contribution of these variants to the proband’s phenotype is unclear. Similarly, BGR 3 has phenotypes frequently reported in patients with *ASH1L* variants including borderline cognitive functioning, ASD, mild bilateral sensorineural hearing loss, and epilepsy. BGR 3 also has the novel phenotype of an abnormal gait. Genetic testing for BGR 3 also indicated three missense variants classified as VUSs in *MED12, POU4F3,* and *ATP2B2*. Of note, this person has hearing loss, and *POU4F3* has an established relationship with hearing loss; in addition, *ATP2B2* has been implicated in hearing loss [32]. *MED12* is implicated in neurodevelopment; however, this particular variant has low pathogenicity predictor scores and is not a conserved residue. At this time, the contribution of these variants to the proband’s phenotype is unclear. 

Like studies of other neurodevelopmental disorders, this study was limited by reporting only on the phenotypes assessed. Other phenotypes could have gone unreported due to their subclinical nature or due to not being assessed at all. However, unlike the previously reported cases, BGR participants have standardized assessments and data collection (RNAP, her, and the GenomeConnect Health Survey) which can capture features that may not have been assessed for in published cases [21]. For example, for many published probands, it is unclear whether dysmorphic features were not assessed or if they were just unreported. However, for BGR probands 1 and 2, dysmorphology was systematically assessed and reported as negative. As a result, we can more appropriately assess the prevalence of dysmorphisms among probands. Standardized assessments of neurocognition and systemic features from the RNAP aherEHR, respectively, help us to better understand gene–disease relationships, the pathogenicity of variants, and the full breadth of the phenotypic spectrum. Combined with the ability of the BGR and GenomeConnect to recontact patients for additional information, our enhanced understanding of the phenotypic spectrum may in turn directly impact clinical care [21].

In summary, we identified three novel LoF *ASH1L* variants in three affected probands that presented with previously unreported phenotypes. Overall, these cases further expand the genotypic and phenotypic spectrum of *ASH1L*-related SCND and demonstrate the value of paired variant and standardized phenotypic data. In the future, clinicians should complete standardized phenotypic assessments and engage families in reporting so that the broader community has a better understanding of the phenotypic spectrum of any given disease.

## Figures and Tables

**Table 1:** Summary of phenotypes previously reported in the literature with previously unreported BGR cases. The denominator for total frequency is determined by the number of previously published probands and BGR cases that commented on the specific phenotypic feature. If a paper did not comment on a phenotype, it is not included in the denominator. (*) indicates that the presence or lack of the given phenotypic feature was reported by the proband’s family via GenomeConnect health surveys. Cases in the literature were included in this table only if they reported detailed phenotypic data [6,8,11,13,14,15,16].

	Total Frequency	BGR 1 (14y M)	BGR 2 (8y M)	BGR 3 (15y M)
*ASH1L* Variant (NM_018489.3)		c.1603G>T p.Gly535*	c.4902_4903del, p.Ser1635Cysfs*18	c.4909C>T, p.Gln1637*
Inheritance		*De novo*	*De novo*	Unknown
Sex	12 Male/3 Female	Male	Male	Male
Type of variant		Nonsense	Frameshift	Nonsense
ID/DD	15/16	+	−	+
ASD	6/10	+	+	+
Motor delay	6/12	−	+	NR
Speech delay	6/10	+	+	NR
Feeding difficulties	4/8	−	+	NR
Seizures	6/11	+	−	+
Obsessive behaviors	4/5	+	+	NR
ADHD or hyperactivity	6/7	+	+	+
Hypotonia	4/8	−	−	+
Genital malformations	5/9	− *	− *	NR
Sleep disorders	6/9	+	−	−
Pectus carinatum/excavatum	2/7	−	− *	NR
Scoliosis	2/7	−	− *	NR
Dysmetria/ataxic gait	2/4	−	+	+
Hyperopia/Astigmatism/Strabismus/Nystagmus	6/10	+	−	−
Hearing impairment	3/8	−	−	+
Gastrointestinal disturbances	6/8	+	+	NR
Anxiety/Depression	2/4	+	−	+
Facial dysmorphisms	8/10	−	−	thin lips
			Additional variants found in *TSHZ3* and *TNR.*	Additional variants found in *MED12, POU4F3*, and *ATP2B2.*

## Data Availability

The original contributions presented in the study are included in the article/Supplementary Materials, further inquiries can be directed to the corresponding author/s.

## Supplementary Materials

The following supporting information can be downloaded at: https://www.mdpi.com/article/10.3390/genes15040423/s1, Table S1: Genotype and phenotype of previously published *ASH1L* probands, for whom phenotypic features were detailed, and three additional BGR-derived subjects. Variants in ASH1L have been reported in large cohort studies and in case reports. If no additional phenotypic information was reported, they are not included here. If a paper did not comment on a specific phenotypic feature, that feature is listed as not reported (NR). (*) indicates that the presence, or lack thereof, of the given phenotype was reported by the proband’s family via GenomeConnect health surveys. An additional variant (c.6238G > A; p.Val2080Ile) has also been reported in Wang et al. [5], but a high frequency in gnomAD (allele frequency 0.00006010, gnomAD v2.1.1) [10], raised doubts about its pathogenicity, and therefore, it is not included in this table.
